# Developing a toolkit to support parents’ involvement in child death review: an experience-based co-design study

**DOI:** 10.1136/archdischild-2024-327642

**Published:** 2024-12-08

**Authors:** Joanna Jane Garstang, Jenna Spry, Gayle Routledge, Anna Pease, Karen L Shaw, Sara Kenyon

**Affiliations:** 1 School of Nursing and Midwifery, University of Birmingham College of Medical and Dental Sciences, Birmingham, UK; 2 Children and Families Division, Birmingham Community Healthcare NHS Foundation Trust, Birmingham, UK; 3 University of Bristol, Bristol, UK; 4 University of Birmingham, Birmingham, UK; 5 A Child of Mine, Stafford, UK; 6 Population Health Sciences, University of Bristol, Bristol, UK; 7 Institute of Applied Health Research, University of Birmingham, Birmingham, UK; 8 Institute of Applied Research, University of Birmingham, Birmingham, UK

**Keywords:** Mortality, Palliative Care, Health services research, Paediatrics

## Abstract

**Background:**

Understanding why children die is important for grieving parents and for informing system improvements aimed at prevention and future care. Many countries have child death review (CDR) process, but little is known about how best to engage parents. The aim of this study was to use experience-based co-design to create a toolkit to support parental involvement in CDR.

**Methods:**

A survey of English paediatric intensive care units (PICUs) and palliative care services explored practices and identified a diverse sample of sites for professional interviews. Bereaved parents were recruited through charities, hospitals and social media. Semistructured interviews were held with parents and professionals followed by co-design workshops to develop the toolkit.

**Results:**

There were 29 survey responses, 13 out of 21 from PICUs and 16 out of 34 from palliative care.

21 multidisciplinary healthcare professionals were interviewed.

23 bereaved parents of children who died aged 0–18 years in 2021–2022, either during hospital admission or under palliative care were, interviewed.

10 parents and 23 professionals participated in co-design meetings. Key emotional touchpoints identified from parents’ experiences were: becoming aware of CDR meetings, being asked for input, knowing the date and receiving feedback. All agreed on the importance of involving parents, with clear communication, and need for resources and training for key workers.

The toolkit includes training videos, a standardised pathway including template letters, feedback form, easy-read leaflet and an animation explaining the importance of involving parents.

**Conclusions:**

Co-design has successfully supported the development of a toolkit of resources in a sensitive area. It required considerable support from bereavement support organisations and researchers. Future evaluation is required.

**Trial registration number:**

ISRCTN14790455.

**WHAT IS ALREADY KNOWN ON THIS TOPIC**

- Many countries have child death review (CDR) programmes to learn from child deaths and help prevent future deaths, but parents are rarely asked to contribute information to reviews or receive feedback afterwards.
- Most bereaved parents will want detailed information about what led to their child’s death, as understanding is an important part of the grieving process.
- Current guidance on CDR does not explain how to involve parents; therefore, key information may be missing from reviews, limiting potential learning from deaths.

**WHAT THIS STUDY ADDS**

- Even when a child’s death is expected, for example, after chronic illness, many parents will still have questions and want to be involved in CDR.
- Parents need a structured process to enable them to share feedback and questions for CDRs; this needs to be alongside bereavement support.
- Bereavement key workers often did not understand their role or the importance of enabling parents to contribute to CDRs.

**HOW THIS STUDY MIGHT AFFECT RESEARCH, PRACTICE OR POLICY**

- This study developed several tools to support parents’ involvement in CDRs, including a well-defined process for regular contact with bereavement support key workers, template letters, feedback forms and an easy-read leaflet.
- Although the toolkit was developed based on the English CDR system, the principles should apply internationally, and the toolkit potentially could be adapted to support CDR in many countries.

## Introduction

 The loss of a child is one of the most traumatic life events for parents^1^ being contrary to normal life order.^2^ Each year in England, around 3500 children die before their 18th birthday, about one-third of these deaths are from chronic conditions, malignancies, congenital abnormalities, genetic disease or following a severe acute illness.^3^ Although in these situations, the diagnosis is usually established prior to death, bereaved parents may still have questions about their child’s illness and treatment. Understanding why their child died is a key need for parents,^4^ making sense of loss is an important part of grieving and those who cannot do so have more profound grief.^5^ Child death review (CDR) addresses this by aiming to understand the reasons for each child’s death, learning from these to prevent future deaths alongside supporting families. CDR programmes vary internationally, most originally focused on unexpected deaths, but the American Academy of Paediatrics (AAP) has recently stated the importance of reviewing deaths including those of children with chronic illness and disability.^6^

Many countries now have CDR programmes; the WHO published CDR guidance aimed particularly towards lower-income and middle-income countries as a mechanism to improve paediatric healthcare.^7^ The WHO CDR aims include ‘that families know that their child’s life was valued, the death is being taken seriously and healthcare workers are committed to learning and improving their practice’, but the CDR process detailed does not involve obtaining information from parents or sharing CDR findings with them.

In England, there is a statutory CDR process^8^; multiagency holistic CDR meetings (CDRM) are a key part, these are held some weeks after the death by the healthcare trust caring for the child. CDRM are attended by professionals only, to enable open discussion. Parents should be informed about the CDR, be able to contribute feedback and receive outcomes, but national guidance lacks detail on how to achieve this. All bereaved families should be allocated a key worker, who acts as a single point of contact between parents and the CDR process, and signposts parents to other services such as bereavement support.

Parents have unique insights into their child’s illness and treatment that professionals may not be aware of.^9 10^ There have been several enquiries into avoidable infant and child deaths,^11^,^14^ which commonly found clinicians ignored parents’ concerns about care, failed to investigate deaths thoroughly and communicated poorly with families. A Japanese study of CDR for children with complex medical conditions dying at home, reported that parents were unable to manage equipment failures and this contributed to several deaths; however, parents’ accounts of events were not included in reviews.^15^ Enabling bereaved parents to ask questions about their child’s illness and share any concerns about healthcare should improve the quality of learning from deaths and families’ experiences of bereavement support.

The Perinatal Mortality Review Tool (PMRT), used for reviewing stillbirth and neonatal deaths, has guidance on how to involve parents.^16^ Similarly, there are detailed guidelines for supporting families following sudden, unexpected child deaths.^17^ These do not cover anticipated deaths of children that occur in hospital or palliative care services, which limits learning from these deaths and support for parents.

The overall aim of this study was to understand existing CDR practice by exploring the views of parents and health professionals and to codesign a best-practice toolkit to support parental involvement in CDR. This paper reports the findings of the co-design work, while detailed analyses of parent and professional experiences will be reported separately.

## Methods

### Objective

The objective was to co-design a best-practice toolkit to support parental involvement in CDR.

We used experience-based co-design methodology (EBCD)^18^ and a survey. EBCD is a well-established process which uses service user and clinicians’ experiences collected using qualitative methods to jointly redesign services. We chose co-design as this should help ensure that the final toolkit meets the needs of bereaved families as well as providing a practical solution for the difficulties healthcare professionals face in CDR. A toolkit designed by professionals alone may not address what is most important for bereaved families, and one designed by families alone may not take account of the realities of service delivery. It was felt that combining these different sets of expertise would produce new knowledge and solutions, grounded in both lived and professional experience.

The protocol is available at https://doi.org/10.1186/ISRCTN14790455.

### Research team and study oversight

The research team consisted of JJG (researcher and Designated Doctor for Child Death), SK (mixed methods maternity researcher), JS (research fellow), AP (research fellow in infant death), KLS (qualitative researcher in palliative care), AMA (paediatric intensive care unit (PICU) consultant) and GR (bereaved parent) to provide a range of perspectives. We were assisted by a stakeholder group with representatives from several bereavement support charities. Bereaved parents were involved in all aspects of the study including design, management, data analysis and toolkit development.

### Survey

We undertook a survey to explore current practice in implementing the 2018 CDR Statutory Guidance^8^ and involving parents in CDR; identifying best practice, challenges and facilitators. The results of the survey were also used to purposefully sample services for professional interviews. Further information on the survey is available in the online supplemental information.

### Co-design

**Figure 1:**
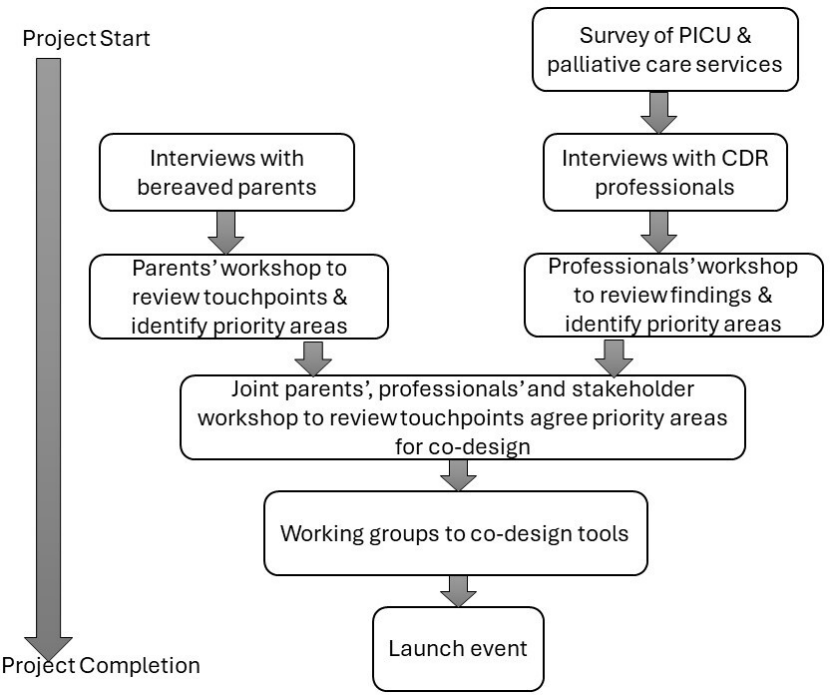
Co-design methods. CDR, child death review; PICU, paediatric intensive care unit.

Our EBCD methods consisted of semistructured interviews with bereaved parents and CDR professionals, with workshops to review findings and co-create solutions. The methods are summarised in figure 1.

### Parental interviews

We advertised the study nationally to bereaved parents directly via social media, through charities and healthcare bereavement teams. Interested parents were sent study information and had telephone conversations with the researcher prior to agreeing to take part. Interviews were held online via Microsoft Teams, face to face at the family home or by telephone between January 2022 and April 2023. Interviews were audio recorded and transcribed.

Bereaved parents (birth parents, step-parents and adoptive parents) were eligible to take part in the study if their child had died between January 2021 and December 2022, during hospital admission, or under palliative care at home or in a hospice. This time period ensured current CDR practices, following the COVID-19 pandemic. Children could have died at any age from birth to 18 years, we excluded infants who died in neonatal care units. Interviews focused on parents’ experiences of CDR and how this could be improved.

### Professional interviews

Using the survey, we identified a purposive sample of PICU and palliative care services. We asked lead CDR clinicians to identify relevant multidisciplinary professionals, supplemented through the snowball sampling method. This included medical consultants, clinical governance staff, nurses, advanced clinical practitioners, bereavement support staff and CDR co-ordinators. We emailed professionals with study information and arrange interviews. All interviews were conducted using Microsoft Teams, were audio recorded and transcribed and took place between April 2022 and September 2023. Interviews focused on professionals’ experiences of involving parents in CDR and barriers or enablers for this.

### Data analysis

Alongside formal qualitative analysis, we identified key ‘touchpoints’, in parents’ CDR experiences. Touchpoints are a vital element of EBCD, described as ‘the key moments and places … where people come into contact with the services and where their subjective experience is shaped, and therefore where the desired emotional and sensory connection needs to be established’.^19^ We considered the touchpoints as key contacts between parents and professionals which shaped parents understanding and involvement in CDR. JS and JJG initially identified touchpoints, which were subsequently agreed with the whole research team.

### Codesign meetings

Separate online workshops were held for parents and professionals who participated in interviews in October 2023 to share findings. At the parents workshop, we agreed touchpoints that would be presented at the later joint meeting and discussed what they felt was most important in parents CDR journeys and what needed to change. Both workshops identified priority areas that needed improvement for the toolkit to address. The workshops were supported by an artist who created visual minutes to illustrate discussions, an example of these is shown in online supplemental image 1. We took notes during these meetings and creating a meeting summary to inform the combined workshop.

We held a third combined workshop of parents, professionals and key stakeholders, facilitated by all the research team in November 2023. We presented the parents’ touchpoints using written quotes. The group considered different tools to support parental CDR. We then formed smaller working groups to develop the tools, meeting several times over the next 3 months, each group had at least one parent, a bereavement support representative, a healthcare professional and researcher.

## Results

### Professionals survey and interviews

There were 29 survey responses, 13 out of 21 from PICUs, response rate 62%, and 16 out of 34 from palliative care, response rate 28%.

We selected three PICU and two palliative care services as sites for professional interviews, choosing sites with different CDR practices to obtain a wide range of experiences. In total, 21 professionals were interviewed, between two and six at each site; ten undertook the role of key worker.

Further information relating to professional survey and interview results can be found in online supplemental information.

### Parents interviews

We were contacted by 38 bereaved parents, of whom 26 were eligible for interview. 23 bereaved parents took part in 21 interviews, relating to 22 children who had died. Details are shown in table 1.

**Table 1:** Details of participating parents and their children

Category	Description	Number
Interviewee	Mother	16
	Father	3
	Both parents	2
Age of child	0–4 years	10
	5–11 years	7
	12–17 years	5
Sex of child	Female	11
	Male	11
Ethnicity of child	White	12
	Black or Black British	3
	Mixed	1
	Other	2
	Not known	4
Location of death	Home	6
	Hospital	10
	Hospice	6
Cause of death	Chronic illness or disability	15
	Short illness	4
	Cancer	3

### Touchpoints and key findings

We identified four main touchpoints from the parental interviews, with two supporting principles identified from parents’ and professionals’ interviews. The touchpoints were agreed at the parents’ workshop. Professional interviews identified the importance of good bereavement support. Touchpoints and interview findings are shown in table 2.

**Table 2:** Touchpoints and key interview findings

Touchpoint	Description	Quotes
Becoming aware of the CDRM	It was very important how CDR was explained to parents, some only read about it in leaflets, others had telephone calls from professionals that they had never spoken with before.	“I think there’s something very cold and impersonal about having a leaflet. Having those sort of honest conversations with a real human being, I think they give you a lot more comfort and they give you a lot more involvement.” Parent 12“And then we got this random call from this random person, who said, “I'm from child bereavement.” We went, “Sure. Okay. Right. Fine.” “Oh, and I'm going to be representing you at this review.”” Parent 16
	Not all parents were aware of the CDR process, for some this was not important, but others felt abandoned and that they were no longer valued as their child had died.	“You’re inundated with so many professionals during that child’s life, that child dies and it’s like ‘tara then’. It’s like you’ve dropped off the scales, and it’s a massive shock to the parents because sometimes they want to ask questions…” Parent 24“…That upsets me, but no one’s told me about feeding that back to anybody. I did say to one of the doctors about it, but I didn’t take it any further because I didn’t know that you could. I didn’t even know that you could provide feedback. It was never said to me.” Parent 22
Being asked for input	Parents all found it very difficult to provide feedback or ask questions for the CDRM as there was no format or structure to do so. Many were discouraged by the lack of guidance.	“’Your feedback would be welcome.’ And I think that’s the problem, and that’s been the frustration as we have gone through the process, that, effectively, you're just given a blank bit of paper, which is nonsensical.” Parent 16“And she said, oh can you put it in an email. I was just like oh my God you know how long it’s taken me to actually pluck up the courage to call you and now you’re saying you want me to put it in an email, okay. So then I tried to write an email and…I got myself all muddled,… I rewrote it about twelve times.” Parent 08
	Parents also found it difficult to ask questions in a non-judgmental manner when they had previously had good relationships with their child’s care team, often for many months or years.	“I was happy to be able to ask some questions. I found it quite difficult because I didn’t want to be blaming anyone, I just had questions. So I found that balance quite difficult…” Parent 06
Knowing the date	Parents did not necessarily know the date on which the CDRM was to be held, but this date often caused further anxiety in the lead-up to it, and distress if the meeting needed to be postponed; this was compounded by poor communication.	“I was thinking, ‘Hang on a minute. This has been eight months. We should have heard by now.’ I was really horrified when [they] said, ‘Yeah, it’s happening on 7th September.’ I said, ‘No one told me.’ We’ve fallen through the gap…” Parent 23“it’s taking a really long time, there’s just no communication there. Like if the meeting was going to be that late I’d just think you know what just send an email and say, I know I told you it was going to be in a couple of weeks but actually you know what we can’t get diaries together.” Parent 08
Receiving feedback	Few parents received feedback after the CDR meeting, but most wanted feedback.	“They never gave us any feedback on it or anything. I think it would’ve been nice… Because there was some feedback that we gave that could detrimentally impact another family…” Parent 19“Much the same as you get reports all the way through every appointment your child has, when they're alive, and so, actually, you kind of feel like, well, maybe there should be a final one.” Parent 14
Supporting principles		
Communication and building relationships	Good communication was key to building relationships with families, enabling them to participate in CDR.	“I just I didn’t want it to be like, oh right tick, we’ve sent that, that’s gone out to that family that’s okay, you know, tick, oh you contacted them tick like we’ve now told them about that. But actually I think it’s that relationship isn’t it…” Parent 08
Bereavement care and support	Parents wanted good bereavement care and support; many felt isolated from services and support after their child had died.	“when a baby’s born, you have midwives and health visitors. It’s the happiest time of your life and you have scheduled day(s)… Why you don’t have the same bereavement care when your world’s falling apart…that would be so beneficial for families when they’re in survival mode. They’re in absolute shock, devastation.” Parent 18“I firmly believe is that our bereavement team hold the families very much in terms of supporting them through that process and any barriers that they encounter as well as navigating through there…on reflection, they’re probably the most key aspects to make sure that these families are supported, and each one is supported in a bespoke manner because their needs will be slightly different…” Professional 21

CDR, child death review; CDRM, child death review meetings.

### Workshops and co-design findings

Eight parents attended the parental workshop. Four professionals attended the initial professional workshop, and a further 30 attended an additional workshop held by the Association of CDR Professionals. Parents’ and professionals’ priorities were very similar and are shown in table 3.

**Table 3:** Parents and professionals priorities for action

Parents’ priorities	Professionals priorities
All families to have access to key workers	Equitable service for all bereaved families
Training for key workers	Training for key workers
Resources for key workers	Adequate resources to conduct reviews and support families
Proactive bereavement support for families	

10 parents and 23 professionals, including 8 key workers, attended the joint parents and professionals’ workshop and agreed priorities for further action. All parents and four professionals had taken part in interviews. There was complete consensus on the value of involving parents in CDR with further discussions highlighting that parents’ information improved the quality of learning from deaths. The role of the key worker was considered and despite significant experience in supporting bereaved families, it became apparent during interviews and workshops that key workers often had little knowledge of CDR. There should be consideration of the most appropriate person as key worker for each family, rather than automatic allocation based on the location of death. This is particularly important when children die in PICU, as this may be at a hospital some distance from home and families may have existing good relationships with local healthcare staff particularly following a long period of ill health.

At the joint workshop, having agreed priorities for change, we broke into three groups to discuss potential solutions or tools to address for each priority. The groups considered key worker training and support, template documents and parental communication pathways, and raising professional awareness of the importance of involving parents in CDR. Participants chose which group to join. At the end of the joint workshop, groups had decided which tools they would create and who would continue working on the codesign process. Co-design groups met several times online over the next 3 months to develop the tool kit, with further rounds of email comments. All the tools were created by the co-design participants although the National Child Mortality Database (NCMD) provided technical support for the animation, and the easy-read leaflet was developed with professional assistance.

### Co-design outputs

1. Most tools were designed for key workers as they have most contact with parents, although all CDR professionals need to be aware of the value of parental involvement. The tools are free to use and available online at https://www.ncmd.info/guidance/parents-cdr-toolkit/a, along with role description for key workers.
2. Key worker training videos explaining their role in CDR.
3. A standardised pathway for engaging with parents, including proactive bereavement support and suggested communications with parents about CDR, a parental feedback form and easy-read leaflet.
4. An animation for professionals explaining the importance of involving parents in CDR.

The pathway for engaging with parents shows minimum suggested contacts and mainly focuses on communication around CDR, recognising that some families may want no involvement with CDR processes. Some may require further additional contact for bereavement support outside of the pathway. Template letters support the pathway, the intention is that the content should be discussed with families first with the letters used to summarise issues. Parents were clear they disliked the phrase ‘child death review’ and preferred terms such as ‘your child’s review’ to be used. The pathway is shown in online supplemental figure 2.

The questions from the feedback form are shown in table 4.

**Table 4:** Questions on feedback form

	Question or comment
1	**Is there anything about your child’s care that you still have questions about?**This could be about medicines, procedures or treatment plans. It could be about decisions that were made to do or not to do something.
2	**Is there anything you found particularly difficult or challenging about getting the care or treatment your child needed?**This could be about accessing the services you needed, for example, specialist doctors or finding out why your child was unwell. It could also be about how information was shared with you or how you were involved (there is another box below that is specifically about these things if you would like to say more).
3	**How well did healthcare professionals communicate with you throughout your child’s care?**Did you understand what you were told? How was the information shared? Was this helpful? Did you feel involved in decisions being made about your child’s care?
4	**Do you have any questions about the care your child received towards the end of their life and when they died?**They might be about timing, medicines, location, who was involved in their care. It might also be about decisions that were made.
5	**Is there anything you would like us to know about what you felt went well during your child’s care?**This could be about people or situations.
6	**Is there anything else that you would like to tell us or ask us that isn’t included in the other questions?**

## Discussion

This study co-designed tools to improve parental involvement in CDR; interviewing 23 bereaved parents and 21 professionals, with a total of 33 people involved in developing solutions. We identified key emotional touchpoints in parents CDR journeys, becoming aware of CDRM, being asked for input, knowing the date and receiving feedback. Communication, good relationships with their key worker and bereavement support were the foundation of parents having more positive CDR experiences. Although key workers are highly skilled in providing bereavement support, many did not understand their role within CDR and did not know how to represent parents at CDRM. Our co-design process developed several tools to support parental involvement.

We could not have completed the study without help from our bereavement support organisations promoting the study to families, attending workshops and participating in the codesign. The low response rate to the professional survey was due to difficulty in identifying palliative care teams who conduct CDRM as there is no national list. We relied on Child Death Overview Panels (CDOP) informing us of their local contacts, and only half did so. We extended the study duration due to recruitment delays.

Despite this, we were able to recruit a wide range of bereaved parents, whose children died from several different disease processes and with varying involvement in CDR. By interviewing healthcare professionals from organisations with different CDR practices, we were able to learn of good practices as well as the difficulties faced trying to support families and follow statutory guidance. This breadth of experience of involvement in CDR and broad expertise of our stakeholders should help ensure that our findings are valid. The project would not have been completed without a research team to drive it forward as with many co-design projects, enthusiasm and attendance at meetings was challenging to sustain. We supported engagement with the co-design process by obtaining email comments on the tools and having them further reviewed by several bereavement support organisations. Given the robust co-design process, the toolkit should represent best practice and be appropriate for use for child deaths from a range of causes. To our knowledge, this is the first co-designed process for supporting parental involvement in CDR, and as such this toolkit should represent current best practice.

EBCD is a well-established process within healthcare research.^18^ Many EBCD projects have included steps such as non-participant observation or using film interviews with participants to highlight touchpoints.^20^ The former would have been impractical given the subject and many bereaved families may not have taken part if filming interviews was required; despite this, our project compares well against many other EBCD projects.^20^ Most EBCD projects aim for local service improvement, we have gone further developing a toolkit for use nationwide. We tried hard to ensure that we followed the principles of co-design keeping the bereaved parents central to all aspects of the project, being involved in designing all elements of the study and contributing to our planned celebration and launch event. We were aware of the power imbalance between the research team, professionals and bereaved parents^21^; and tried hard to address this by including all relevant perspectives, developing shared understanding, reciprocity and maintaining relationships. Our project shows that co-design can be used successfully with vulnerable participants, other co-design projects have used trauma-informed approaches for participants with complex needs.^22^

We had intended to hold face to face workshops, but parents expressed a clear preference for these to be online with professionals. We were concerned about how to offer emotional support if required and power imbalances with this format. To mitigate this, parents joined the joint workshop in advance of professionals, and a bereavement support organisation representative was available by telephone throughout the meeting and afterwards if parents needed. Parents were also advised they could leave the meeting at any point and rejoin again if they wished. We held most discussions in small groups with representatives from bereavement support organisations as facilitators. We offered regular contact to parent participants and met with them outside of co-design meetings as needed.

Our finding that parents want to be involved in the review of their child’s death concur with those of parents following perinatal loss, and our tools are similar to the pathway for supporting parental engagement in PMRT,^23^ but are designed to be applicable for a much wider range of child deaths, as well as covering training and raising awareness. Participants felt it was vital that commissioners, managers and senior professionals in CDR understood the importance of involving parents, so we developed a short animation aimed for them. This is similar to other projects that have produced illustrated literature for professionals telling them of parents’ experiences of infant loss.^24^

Although the toolkit was developed for use with the English CDR system, the tools could be used to support parental involvement in CDR internationally, as there is limited practice and guidance in this area. Japan is currently developing CDR, and has identified the lack of guidance in how to include families as a barrier.^25^ The AAP CDR statement promoted the role of paediatricians in communicating with bereaved families about CDR.^6^ The feedback form could easily be used to enhance communication with relatives after deaths of patients at any age. This toolkit will need future evaluation to see if it improves parental involvement in CDR and to establish its acceptability and effectiveness for a wider population of bereaved parents and clinicians. The toolkit has potential to lead to greater learning from deaths and help prevent future child deaths.

## Supplementary material

10.1136/archdischild-2024-327642 online supplemental file 1

10.1136/archdischild-2024-327642 online supplemental figure 1

10.1136/archdischild-2024-327642 online supplemental figure 2

## Data Availability

Data are available on reasonable request.
