# Supplementary material for: Developing a toolkit to support parents’ involvement in child death review: an experience-based co-design study
Source: Arch Dis Child. 2024 Dec 8;110(4):e327642. doi: 10.1136/archdischild-2024-327642 (PMC12013563; doi:10.1136/archdischild-2024-327642)
Supplement: online supplemental file 1 [file archdischild-110-4-s001.pdf]

SUPPLEMENTARY INFORMATION

Professional Survey Methods

As most inpatient child deaths occur in level 3 Paediatric Intensive Care Units (PICU), who care for the most complex children; we sent a survey to all 21 English Level 3 PICU through the Paediatric Critical Care Society, asking for it to be completed by the lead clinician responsible for mortality review. We did not include neonatal intensive care units (NICU) as the study excluded neonatal deaths. To identify palliative care services, supporting children who die at home or hospices, we emailed all 58 Child Death Overview Panels (CDOP) asking for details of lead clinicians for palliative care CDRM. We sent a survey link to this clinician. Surveys were distributed between November 2022 and March 2023.

Professional Survey and Interview Results

The survey was sent to lead CDR clinicians at 21 Level 3 PICU with13 responses, response rate 62%. It was more difficult identifying palliative care services responsible for CDRM as there is no national list. Of the 58 CDOPs emailed, 34 gave us contact details for lead palliative care CDR clinicians; of these 16 responded, giving a total of 29 survey responses, response rate 28%.

The PICU survey was initially sent during peak winter pressures, and the ethical requirement to send the survey through the Paediatric Critical Care Society meant that we were unable to send reminders directly to non-responders.

26 of 29 respondents reported that their service mostly or always or usually held CDRM, 25/29 informed parents of CDRM, and 19/29 mostly or always invited parents to contribute to CDRM. The method of informing parents varied greatly from conversations with keyworkers, personal letters and standard leaflets.

Of the five sites selected for professional interviews, one PICU and one palliative care service had fully implemented the 2018 CDR statutory guidance, held CDR meetings and invited parents' involvement. One PICU had yet to implement CDR, did not hold CDR meetings and parents were rarely involved. The remaining PICU and palliative care service were starting to implement CDR but finding this challenging. The details of professionals taking part in interviews are shown in Supplementary Table 1.

Supplementary Table 1: Details of professionals interviewed.

Job role	Number of interviewees
**PICU consultant**	5
**Community paediatric or palliative care consultant**	3
**Senior or specialist nurse**	6
**CDR co-ordinators**	4
**Bereavement support workers**	3
**Total**	21
