# Supplementary figures and images for: Developing a toolkit to support parents’ involvement in child death review: an experience-based co-design study
Source: Arch Dis Child. 2024 Dec 8;110(4):e327642. doi: 10.1136/archdischild-2024-327642 (PMC12013563; doi:10.1136/archdischild-2024-327642)

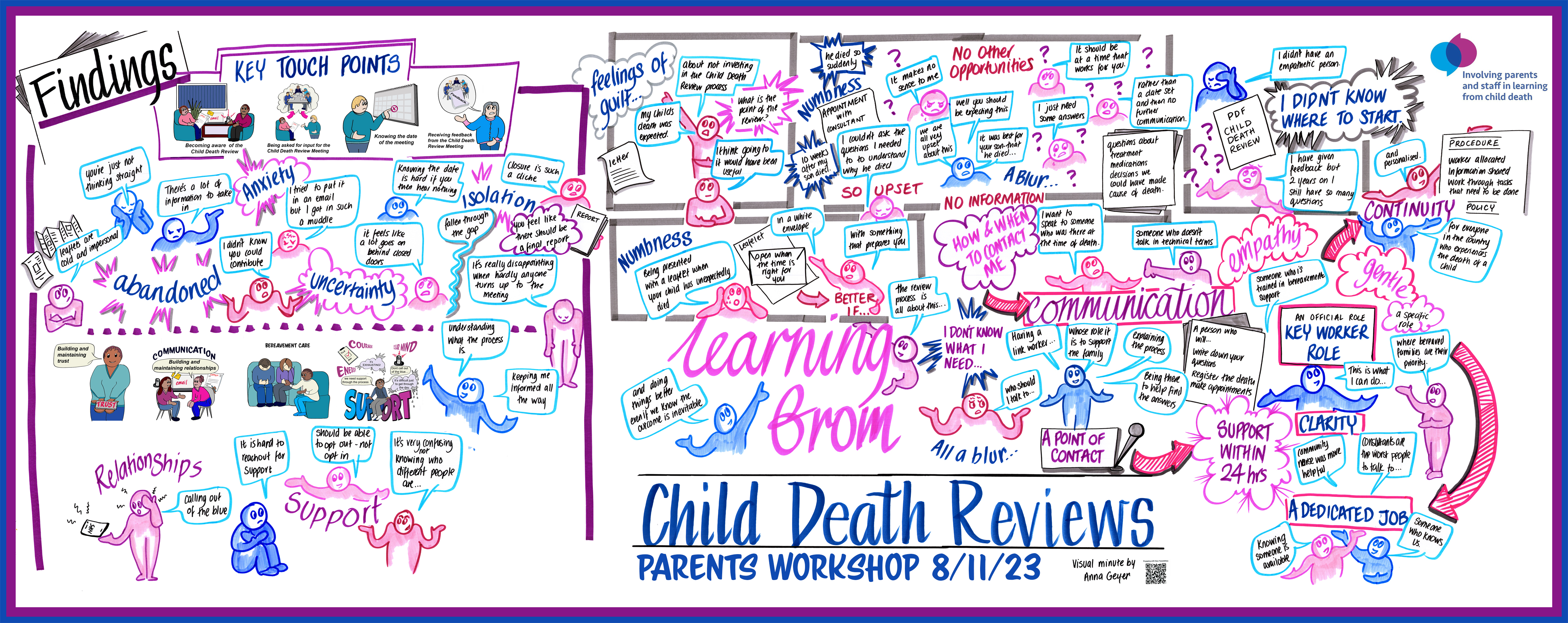

Supplement: online supplemental figure 1 [file archdischild-110-4-s002.jpg]

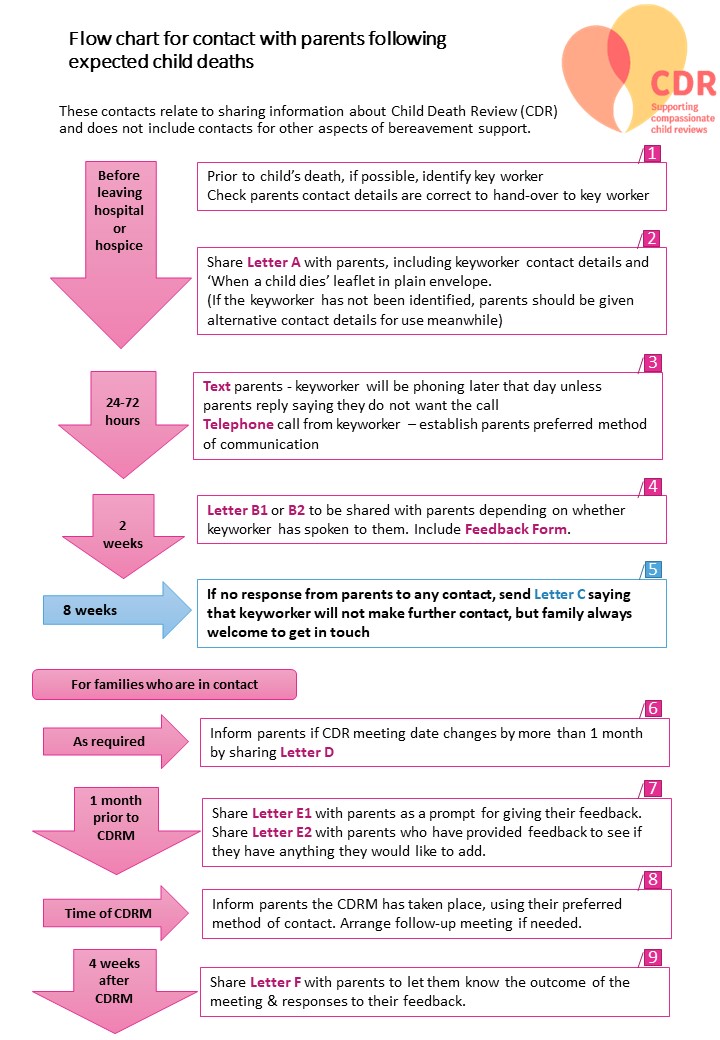

Supplement: online supplemental figure 2 [file archdischild-110-4-s003.jpg]
